# Kinematic Analysis of the Lower Limb in Uchi-Mata: Comparison Between Elite Athletes Specializing and Non-Specializing

**DOI:** 10.3390/jfmk10040378

**Published:** 2025-09-30

**Authors:** Ciro José Brito, Naiara Ribeiro Almeida, Ignacio Roa-Gamboa, Lindsei Brabec Mota Barreto, José Raimundo Fernandes, Lúcio Marques Vieira-Souza, Otávio de Toledo Nóbrega, Alfonso López Díaz de Durana, Bianca Miarka, Esteban Aedo-Muñoz

**Affiliations:** 1 Facultad de Ciencias Médicas, Universidad de Santiago de Chile, Avenida Libertador Bernardo O’Higgins n°3363—Estación Central, Santiago 8320000, Chile; ciro.brito@usach.cl; 2 Postgraduate Program of Physical Education, Department of Physical Education, Federal University of Juiz de Fora, Governador Valadares 35010-900, Brazil; profa.naiara.ribeiro@gmail.com (N.R.A.); ignacio.roa.27@gmail.com (I.R.-G.); mundegv@hotmail.com (J.R.F.); 3 Education Department, Physical Education School, Federal University of Grande Dourados, Dourados 79804-970, Brazil; lindsei@brabec.com.br; 4 Physical Education School, University of Minas Gerais State, Passos 37900-106, Brazil; profedf.luciomarkes@gmail.com; 5 Graduate Program in Medical Sciences, University of Brasilia, Campus Universitario Darcy Ribeiro, Brasilia 70910-900, Brazil; otavionobrega@unb.br; 6 Sports Department, Faculty of Physical Activity and Sports Science—INEF, Universidad Politécnica de Madrid, 28040 Madrid, Spain; alfonso.lopez@upm.es; 7 Laboratory of Psychophysiology, Federal University of Rio de Janeiro, Rio de Janeiro 21941-914, Brazil; miarkasport@hotmail.com

**Keywords:** martial arts, sport performance, biomechanics, kinematics, task performance and analysis

## Abstract

**Background:** Uchi-mata is one of the most frequently used throwing techniques in judo, yet little is known about the kinematic factors distinguishing specialists from non-specialists. This study compared lower-limb kinematics during uchi-mata across its three phases in elite judokas. **Methods:** Forty athletes (12 female, 28 male; 24.5 ± 5.9 years) were classified as specialists (n = 20) or non-specialists (n = 20). Photogrammetry assessed hip, knee, and foot displacement, velocity, acceleration, and timing during the Approach, Turning, and Throw phases. Analyses were performed using mixed-effects models with group, phase, and sex as fixed effects, plus exploratory multivariate tests (*p* < 0.05). **Results:** Specialists executed faster movements in the Approach (*p* = 0.036, d = 0.69) and Throw phases (*p* = 0.010, d = 0.85), showed greater hip displacement during Approach (*p* = 0.008, d = 0.89), and achieved superior knee and foot displacement in Throw (*p* = 0.005 and *p* = 0.003). Final positioning also differed, with specialists displaying higher knee (98.5 ± 14.5 vs. 86.3 ± 17.8 cm, *p* ≤ 0.001) and foot (121.0 ± 19.7 vs. 104.4 ± 27.4 cm, *p* = 0.034) heights, but lower hip position (61.9 ± 4.2 vs. 75.6 ± 7.5 cm, *p* = 0.021). Sex showed no significant effects or interactions, indicating that these group differences were consistent across male and female athletes. **Conclusions:** Uchi-mata specialists demonstrated superior displacement and velocity control, particularly in the Approach and Throw phases, reflecting greater neuromuscular coordination and efficiency. These findings provide practical markers for coaches and athletes to guide training focused on mobility, strength, and technical drills that enhance hip, knee, and foot displacement, supporting the optimization of uchi-mata performance in elite judo.

## 1. Introduction

Elite judokas consistently demonstrate exceptional strength and endurance [1,2], with force platform analyses reporting ground reaction forces exceeding 2000 N during throw execution [3]. In this sense, Ren et al. [2] noted that, although superior strength can offer an advantage, minor technical differences often determine victory when physical capacities are similar. Motion capture studies have quantified these distinctions; for example, elite judokas produce 15–30% higher trunk angular velocities during seoi-nage compared to collegiate athletes [4], and show different joint configurations, with knee flexion angles differing by up to 32° in key phases [5]. Such biomechanical advantages may allow specialists to generate more efficient unbalancing and execution phases. Despite these advances, comprehensive kinematic studies comparing uchi-mata execution between specialists and non-specialists remain scarce, limiting understanding of the technical determinants that distinguish expertise in this widely used throwing technique.

Previous research has identified performance markers separating elite from less experienced judokas. Elite competitors demonstrate higher grip strength [1,2], broader technical repertoires [6], and greater adaptability in attack direction [6,7]. Dopico-Calvo et al. [8] found that athletes excelling in ground fighting display distinct grip techniques. Meanwhile, found that successful ground fighters applied distinctive gripping patterns that facilitated transitions and control. Lech et al. [9] reported that Georgian athletes exhibited greater technical variability than Japanese or French judokas, while Japanese athletes attacked more frequently in the latter half of bouts—highlighting that high-level performance integrates both physical and tactical attributes.

Biomechanical analysis provides objective, quantifiable data to evaluate such differences [10], employing instruments including force platforms [11], and kinematic analysis [4,5,12]. Notably, kinematic analysis has been widely applied to study judo techniques [4,5,12,13], isokinetic dynamometers [14,15], and 3D motion capture systems [4,5,12,13]. These tools not only identify mechanical advantages but also guide targeted training interventions. For example, Choi and Song [12] analyzed seoi-nage in 12 judokas (6 elite, 6 non-elite) using 3D motion analysis, measuring angular velocities of trunk and pelvis rotation, hip flexion/extension, and knee flexion. Elite athletes showed significantly higher values across all parameters, suggesting superior rotational power and lower limb coordination—factors that can inform trunk-strengthening and timing drills in training. Already, Ishii et al. [5] compared seoi-nage execution between three World Championship medalists and nine collegiate athletes using an 18-camera VICON system at 250 Hz. Variables included pivot leg knee angle, shoulder rotation, and swing leg shank angle. Elite judokas maintained significantly greater knee flexion during critical phases (e.g., 114.6° ± 11.0 vs. 82.7° ± 8.2 in the Kake phase), indicating refined joint control—knowledge that can guide flexibility and joint stability programs.

Analyzing a hip technique, Pucsok et al. [4] examined harai-goshi in 28 athletes (14 experienced, 14 beginners) combining force platform and kinematic analysis. Experienced fighters generated higher horizontal ground reaction forces (1800 N vs. 1200 N) and demonstrated more efficient timing, emphasizing the importance of explosive force and coordinated entry. Specifically, about the uchi-mata analysis, Hamaguchi et al. [16] investigated 20 participants (10 highly skilled, 10 less skilled), measuring center of mass velocity, joint angular velocities, and angular momentum. Skilled judokas achieved significantly greater peak values in all variables, reinforcing the need for drills targeting hip and shoulder rotation speed. Similarly, Zaggelidis and Lazaridis [17] showed that timing precision and vertical ground reaction force magnitude are decisive for uchi-mata efficiency.

Although uchi-mata ranks among the most frequently applied and successful competition techniques [18,19,20], few studies have explored its execution in detail [17,21]. Considering the timing, velocity, acceleration, and displacement of the hip, knee, and foot during uchi-mata, this study aimed to: (a) compare execution of the judo throwing technique across its three phases (Approach, Turning, and Throw) performed by specialist and non-specialist international athletes through both phase-specific and global movement analyses, (b) identify the kinematic predictors associated with technique effectiveness across all phases of execution, and (c) determine whether expertise differences are consistent throughout the complete movement pattern or limited to specific technique components. The findings of this study provide valuable insights for coaches and physical trainers, helping them understand the key biomechanical differences that set specialists apart in this critical attack. By employing both phase-specific and holistic analytical approaches, this research offers a comprehensive understanding of movement characteristics that distinguish expertise, enabling more targeted and effective training interventions. With this knowledge, training programs can be more effectively tailored to enhance the technical aspects that optimize performance, with particular emphasis on movement amplitude control, velocity optimization, and neuromuscular coordination throughout the entire technique execution. We hypothesize that specialist judokas will demonstrate differences during the three uchi-mata phases in their lower limbs compared to those who prefer other techniques, and that these differences will be reflected in both phase-specific parameters and global movement characteristics that distinguish overall technique proficiency.

## 2. Materials and Methods

### 2.1. Experimental Approach

This is a cross-sectional comparative study aimed at analyzing the performance of the uchi-mata technique among international-level judokas. Two groups were assessed: (a) specialists, who identified uchi-mata as their first or second preferred throwing technique (tokui-waza), and (b) non-specialists, who preferred other techniques. Classification was based on the athletes’ responses during an anamnesis interview, in which each participant listed their three most used techniques in training and competition. Performance was evaluated through videophotometry to determine the kinematic behavior of the lower limbs (hip, knee, and foot).

The research process began with one of the researchers contacting the National Judo Federation to present the study’s objectives. Upon receiving approval, the research team reached out to coaches and athletes who met the inclusion criteria. Participants who agreed to participate signed an informed consent form and were assessed in the biomechanics laboratory of the National Institute of Sports. This study was approved by the research ethics committee of the university where the data were analyzed (protocol: 275/2024).

### 2.2. Participants

The inclusion criteria for participants were as follows: (a) being a member of the national team, (b) black belt; (c) ≥18 years old; (d) having participated in international competitions in the previous year (≥South America Judo Championship); and (e) currently training for competition. Exclusion criteria were: (a) recordings of participants affected by environmental conditions, resulting in the loss of one or more phases of the technique; (b) participants who chose to withdraw from the study; (c) athletes with joint instability (shoulder, knee, ankle); and (d) having injuries that would affect uchi-mata performance.

The minimum sample size was determined based on previous kinematic studies in judo [5,17] using Granmo 8.0 software (REGICOR, IMIM, Barcelona, Spain). Next, the minimum required sample size was estimated at 32 participants with a significance level of α = 0.05 (two-tailed) and a statistical power of 0.8. However, an additional eight participants were added to account for potential sample loss. Therefore, the final sample comprised 40 judokas (♀ = 12). The participants were divided into two matched groups: (a) specialists (4 ♀ and 16 ♂), defined as those who use uchi-mata as their first or second preferred technique (tokui-waza), and (b) non-specialists (8 ♀ and 12 ♂), who prefer other techniques as their first and second tokui-waza. Table 1 presents the anthropometric characteristics, age, and weekly training hours, with no significant differences between the groups (*p* ≥ 0.05).

### 2.3. Experimental Procedures

All participants underwent anamnesis before the kinematic test, during which they indicated their preferred side for applying the techniques and their three tokui-waza. Anthropometric measurements were performed by an ISAK Level 2 certified evaluator following International Society for the Advancement of Kinanthropometry guidelines [22]. Body mass measurements were subsequently taken using a Detecto^®^ scale (339, Webb City, MO, USA), height was measured with a Welmy^®^ stadiometer (W2000A, São Paulo, Brazil), and body composition was assessed using the Jackson and Pollock protocol for male [23] and female [24]. Lower limb lengths were measured with a non-elastic anthropometric tape (Sanny^®^, São Paulo, Brazil).

Kinematics of Uchi-Mata—The kinematic analysis was performed using a ten-camera photogrammetry system operating at a capture frequency of 200 Hz. The system was calibrated before each measurement according to Vicon standards, with data residuals adjusted to within 2 mm, as recommended by Merriaux et al. [25]. For this, 16 reflective markers (14 mm) were positioned bilaterally at the following anatomical points on the lower limbs, as per the manufacturer’s guidelines [26]: (a) Anterosuperior and posterosuperior iliac spines; (b) Lower and upper thirds of the lateral surface of the thigh; (c) Lower and upper thirds of the lateral surface of the shank; (d) Lateral epicondyle of the femur; (e) Lateral malleolus; (f) Second metatarsal head; (g) Calcaneus. To ensure measurement accuracy and prevent movement artifacts, all markers were secured directly to the participant’s skin using hypoallergenic double-sided adhesive tape. The throwing athlete (tori) performed the technique without judogi to ensure marker capture accuracy, while the receiving athlete (uke) wore the judogi to maintain ecological validity of the technical gesture.

**Figure 1:**
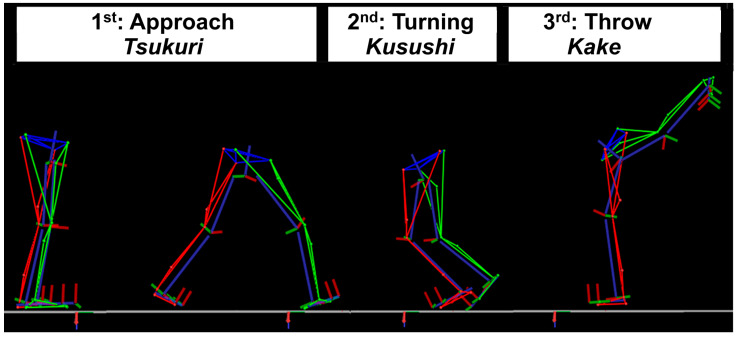
Sagittal view of the photogrammetry analysis of uchi-mata, divided into the Approach (Tsukuri), Turning (Kuzushi), and Throw (Kake) phases. Red represents the left lower limb, green the right lower limb, and blue lines indicate the mechanical axes of the anatomical segments.

For data collection, participants were paired based on body mass, allowing for a tolerance of up to a 5% difference. All participants began with a warm-up that included 5 min of stretching, 3 min of partial uchi-mata attempts (uchi-komi—repetition training), and 2 min of ukemi (break fall practice). Each tori (performer) executed three full repetitions of the technique, with 1-min breaks between attempts. Participants were instructed to perform the classical uchi-mata handgrip (high collar and sleeve). All attempts were performed at maximum speed, directing the throw toward their dominant side. For analysis, the complete movement from the initial unbalancing to the completion of the throw was divided into three distinct phases: (a) Tsukuri—Approach: From the initial movement of the attacking limb to the start of rotation in the supporting limb. (b) Kuzushi—Turning: From the start of the supporting limb’s rotation to the beginning of the attacking limb’s movement. (c) Kake—Throw execution: From the start to the completion of the attacking leg’s motion. Figure 1 illustrates these three movement phases.

**Figure 2:**
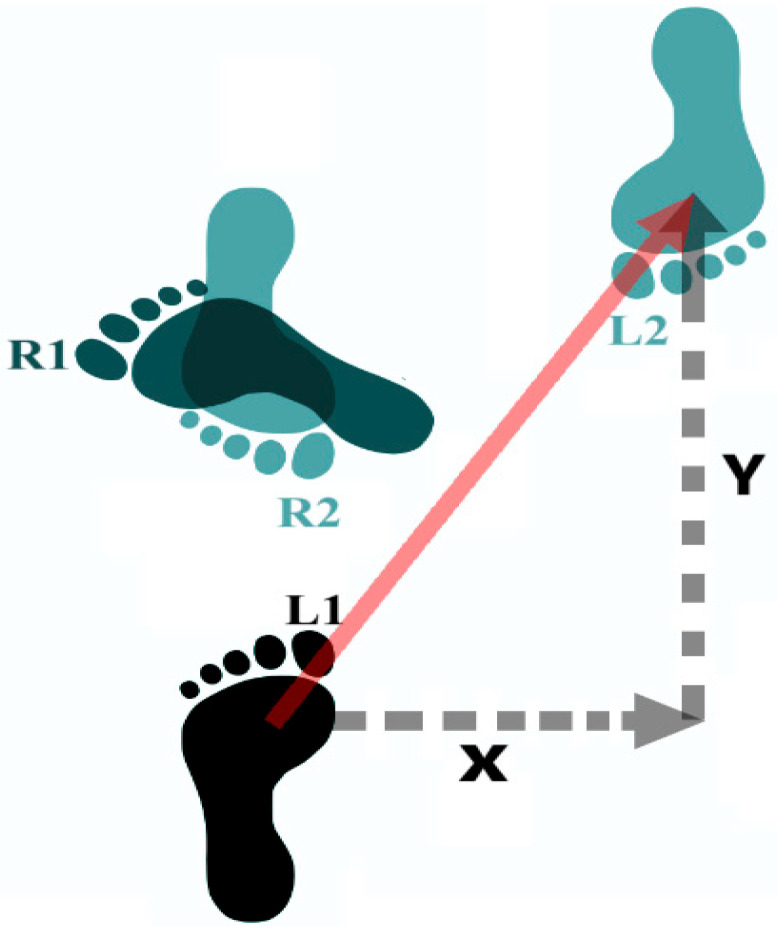
Example of foot displacement in the Turning phase for a right-handed athlete. L1—initial position of the support limb, L2—final position of the support limb, R1—initial position of the attack limb, R2—final position of the attack limb. Red arrow—calculated displacement. This procedure was followed for the hip, knee and foot displacement.

For comparative analysis of specialists vs. non-specialists, the following variables were quantified across the Tsukuri (Approach), Kuzushi (Turning), and Kake (Throw) phases of the technique, in accordance with Windolf et al. [27]: (a) time; (b) linear velocity and acceleration of the foot in the anteroposterior direction (Y axis) during the Approach and Turning phases and vertical direction (Z axis) during the Throw phase; (c) angular velocity and acceleration of the knee and hip during flexion and extension movements and (d) three-dimensional linear displacement of the hip, knee, and foot using the markers of anterosuperior iliac spine, lateral epicondyle and calcaneus, respectively. The displacement of each phase was calculated as follows: (a) Approach phase: Displacement of the attacking limb along the anteroposterior (Y) axis. (b) Turning phase: Resultant displacement of the supporting limb derived from trigonometric relationships between mediolateral (X) and anteroposterior (Y) axis movements (see Figure 2 and Equations below for hip, knee, and foot calculations). (c) Throw phase: Displacement of the attacking limb along the vertical (Z) axis.

Data acquisition and processing adhered to the manufacturer’s protocols [26] using Vicon Nexus software (version 1.8.5; Vicon Motion Systems Ltd., Oxford, UK). Kinematic parameters were sampled at 200 Hz, and all trajectories were filtered using a zero-lag Butterworth low-pass filter (cutoff: 6 Hz).

### 2.4. Lower-Limb Turning (Kuzushi) Displacement Calculation

To quantify joint and foot displacement during the turning phase, the Euclidean distance was calculated separately for the hip, knee, and foot using the following equations:

$$D_{h}\sqrt{\left(Dh_{x}\right)2+\left(Dh_{y}\right)2}\;\;\;\;\;\;\;\;\;\;\;\;\;\;D_{k}\sqrt{\left(Dk_{x}\right)2+\left(Dk_{y}\right)2}\;\;\;\;\;\;\;\;\;\;\;\;\;\;D_{f}\sqrt{\left(Df_{x}\right)2+\left(Df_{y}\right)2}$$

where

- Dh = hip displacement;
- Dk = knee displacement;
- Df = foot displacement;
- x, y = Cartesian coordinates (horizontal and vertical directions, respectively).

Figure 2 provides a representative example of foot displacement during the turning phase, highlighting the trigonometric methodology.

### 2.5. Total Uchi-Mata Analysis

To provide a comprehensive understanding of overall movement characteristics that distinguish uchi-mata expertise, a global movement analysis was conducted by aggregating biomechanical data across all three technique phases. This holistic approach involved pooling measurements from the Approach, Turning, and Throw phases for each biomechanical variable category, creating a combined dataset that represented the complete technique execution profile for each participant. The global analysis examined ten primary movement characteristics organized into four categories: temporal efficiency (execution time), velocity parameters (hip angular velocity, knee angular velocity, and linear foot velocity), acceleration parameters (hip angular acceleration, knee angular acceleration, and linear foot acceleration), and displacement parameters (hip displacement, knee displacement, and foot displacement). For each variable category, individual measurements from all three phases were combined to calculate overall means and standard deviations for specialists and non-specialists.

### 2.6. Statistical Analysis

Data analysis was performed using a hierarchical approach combining multiple statistical methods to comprehensively examine the biomechanical differences between uchi-mata specialists and non-specialists. Prior to analysis, statistical assumptions were tested including normality using the Shapiro–Wilk test, homogeneity of variances using Levene’s test, and linearity of covariate relationships through scatterplot examination. The primary analytical approach employed mixed-effects models to account for the repeated measures structure across the three technique phases (Approach, Turning, Throw) while controlling for anthropometric covariates (age, height, weight, BMI, lower-limb length, and training hours per week). These models included fixed effects for group (specialist vs. non-specialist), sex, phase, and group × phase interactions, with random intercepts for participants to account for individual differences.

Secondary analyses included phase-specific independent *t*-tests to examine group differences within each technique phase, calculation of effect sizes using Cohen’s d to assess practical significance, and multivariate analysis of covariance (MANCOVA) for simultaneous analysis of multiple dependent variables within each phase. The MANCOVA included group as the fixed factor, kinematic variables as dependent variables, and the athletes’ characteristics (sex, age, height, weight, BMI, lower-limb length, and training) as covariates to ensure that observed group differences reflect technical expertise rather than anthropometric or training-related factors.

Additionally, a global movement analysis was conducted by combining data from all three phases to provide a holistic assessment of overall movement characteristics. This analysis involved pooling biomechanical measurements across phases for each variable category (temporal, velocity, acceleration, and displacement parameters) and performing independent *t*-tests to compare specialists and non-specialists on their overall movement patterns throughout the complete technique execution.

Post hoc comparisons were conducted using Bonferroni correction for multiple comparisons when significant main effects or interactions were detected. Statistical analyses were performed using Python 3.11 with statsmodels, scipy, pandas, and matplotlib libraries. Cohen’s d was used to estimate the effect size [28], applying the cut-off points suggested by Yagin et al. [29] for highly trained athletes: trivial (<0.25), small (0.25–0.5), moderate (0.50–1.0), and large (>1.0). The global analysis provided complementary insights into the phase-specific comparisons by identifying overall movement characteristics that distinguish expertise across the entire technique execution, with particular emphasis on movement amplitude and velocity control patterns.

## 3. Results

After adjusting for the covariates (sex, age, height, weight, BMI, lower-limb length, and training), the analysis revealed the following significant differences. The primary analyses were conducted using linear mixed-effects models, which included fixed effects for group, phase of technique, sex, and their interactions, with random intercepts for participants. The LMM revealed significant group differences mainly in temporal efficiency and displacement parameters. Specialists performed faster movements in the Approach (*p* = 0.036, d = 0.69) and Throw phases (*p* = 0.01, d = 0.85), showed greater hip displacement during the Approach phase (*p* = 0.008, d = 0.89), and achieved superior knee and foot displacement in the Throw phase (*p* = 0.005 and *p* = 0.003, respectively). The sex factor did not show significant main effects or interactions with group or phase, indicating that the observed differences between specialists and non-specialists were consistent across male and female athletes.

As a secondary exploratory step, MANCOVA revealed significant differences between specialists and non-specialists across multiple biomechanical variables, with temporal efficiency and displacement patterns emerging as the primary discriminators of difference. Specialists demonstrated superior temporal performance in both the Tsukuri (F = 4.732, *p* = 0.036, d = 0.69) and Kake phase (F = 7.251, *p* = 0.010, d = 0.85). Displacement analysis showed that specialists achieved significantly greater hip during the Tsukuri (F = 7.926, *p* = 0.008, d = 0.89) and superior knee and foot displacement during the Kake (knee: F = 9.002, *p* = 0.005, d = 0.95; foot: F = 10.083, *p* = 0.003, d = 1.0). While angular velocities showed moderate effect sizes favoring specialists, particularly for hip angular velocity in the Kake (d = 0.5), these differences did not reach statistical significance. Detailed kinematic data for lower limb across phases are summarized in Table 2.

**Figure 3:**
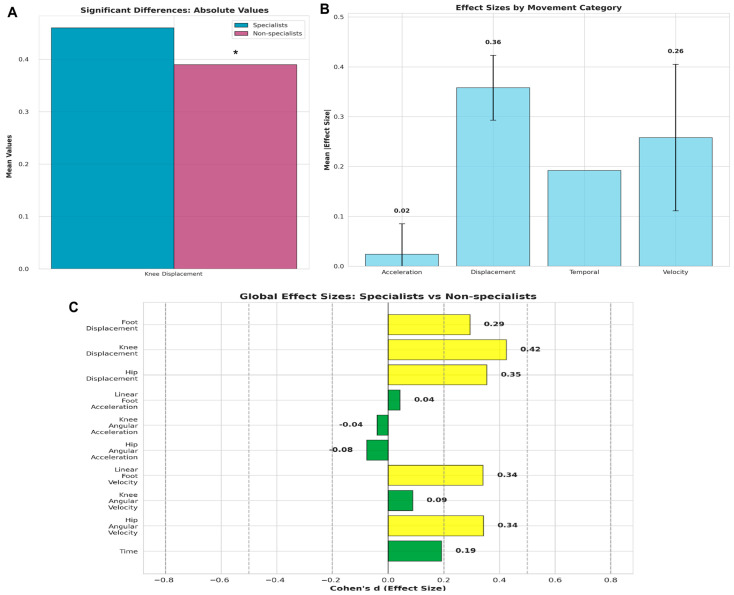
Global movement analysis comparing Specialists and Non-specialists across all technique phases. (**A**) Absolute values comparison for significant variables with statistical markers (* *p* < 0.05); (**B**) Mean effect sizes by movement category showing displacement parameters demonstrate the largest differences; (**C**) Effect sizes for all biomechanical variables with color coding indicating magnitude (yellow = small effect ≥ 0.2, green = negligible effect < 0.2).

The global movement analysis across all technique phases revealed distinct biomechanical patterns that characterize Uchi-mata (Figure 3). When examining the overall movement characteristics, specialists demonstrated significantly greater knee displacement compared to non-specialists (*p* = 0.023, d = 0.42), indicating superior control and range of motion in the attacking lower-limb. Hip angular velocity showed a strong trend toward higher values in specialists (*p* = 0.065, d = 0.34), as did linear foot velocity (*p* = 0.066, d = 0.34), suggesting enhanced rotational and linear movement capabilities. Hip displacement also approached significance with specialists showing greater movement amplitude (*p* = 0.056, d = 0.35). Acceleration parameters showed no meaningful differences between groups, with hip angular acceleration (*p* = 0.681), knee angular acceleration (*p* = 0.832), and linear foot acceleration (*p* = 0.817) all demonstrating negligible effect sizes.

**Figure 4:**
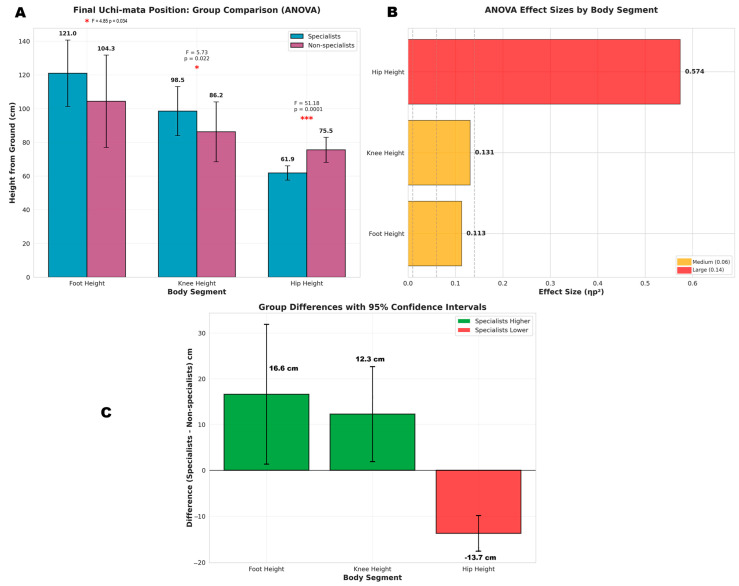
Analysis of final Uchi-mata position comparing specialists and non-specialists. (**A**) shows mean heights (±SD) for foot, knee, and hip positions with F-statistics, *p*-values, and significance markers (* *p* < 0.05, *** *p* < 0.001). (**B**) displays effect sizes (ηp^2^) with reference lines indicating small (0.01), medium (0.06), and large (0.14) effect thresholds. (**C**) illustrates group differences with 95% confidence intervals.

Analysis of the final uchi-mata position (Figure 4) revealed significant differences between the groups. Specialists maintained a significantly higher foot height (F_(1,38)_ = 4.847, *p* = 0.034, ηp^2^ = 0.113) and a higher knee height (F_(1,38)_ = 5.727, *p* = 0.022, ηp^2^ = 0.131). Most notably, hip height demonstrated the most pronounced group effect (F_(1,38)_ = 51.182, *p* ≤ 0.001, ηp^2^ = 0.574), with specialists achieving a significantly lower position than non-specialists. The effect sizes ranged from medium for foot and knee height to large for hip height.

## 4. Discussion

To the best of our knowledge, this study is the first to apply a comprehensive kinematic analysis protocol to compare the execution of uchi-mata among international-level judokas, specifically contrasting specialists and non-specialists of the technique. Our comprehensive global movement analysis revealed that uchi-mata expertise is characterized by enhanced movement amplitude and velocity control rather than maximal power generation. While specialists and non-specialists demonstrated similar acceleration capabilities across all, specialists showed consistently higher velocities, with hip angular velocity approaching significance and linear foot velocity demonstrating a strong trend. Most notably, knee displacement emerged as the only globally significant difference, indicating that expertise is fundamentally distinguished by superior movement control and range utilization throughout the complete technique execution. These technical distinctions underscore that expertise in uchi-mata is not solely defined by velocity but by the precise optimization of range of motion, timing, and final body configuration to maximize throwing efficiency. This offers a nuanced understanding for athletes and coaches aiming to refine performance, highlighting the critical importance of dynamic flexibility, strength, and precise movement sequencing for an effective uchi-mata.

Unexpectedly, despite traditional assumptions that explosive power and acceleration would be decisive factors in differentiating expertise, our results showed no meaningful differences between specialists and non-specialists in acceleration variables. Instead, specialists displayed consistent advantages in displacement and velocity control, reinforcing that neuromuscular coordination and movement efficiency, rather than maximal force production, are central determinants of uchi-mata performance [30,31]. This constitutes a novel contribution by challenging a strength-centered paradigm and suggesting that technical expertise in this throw emerges primarily from optimized motor control. Previous studies examining the technical and tactical aspects of judo competitions have demonstrated that uchi-mata is a frequently employed and highly effective technique [20,32]. One key factor driving athletes to preferentially use this technique is the superior couple efficiency it offers compared to other throwing techniques [33,34]. While uchi-mata is biomechanically straightforward, its successful execution requires a judoka to possess high motor coordination and precise timing [33,35].

The global analysis provides crucial insights into the holistic nature of uchi-mata expertise that extends beyond phase-specific differences. The finding that knee displacement was the only variable to achieve statistical significance when examined across all phases suggests that consistent movement amplitude control is a fundamental characteristic of expertise. This aligns with motor learning principles that emphasize the importance of movement variability and adaptability in expert performance [36,37]. The moderate effect size for knee displacement indicates that this difference is not only statistically significant but also practically meaningful, representing an 18% greater movement range in specialists compared to non-specialists. Crucially, this suggests that knee displacement may serve as a practical biomarker of uchi-mata expertise. To our knowledge, no prior study has identified a single kinematic parameter with such discriminant potential in elite judokas. This variable could therefore be used in technical assessments, progression monitoring, and even talent identification programs.

A kinematic analysis of judo techniques, such as uchi-mata, demonstrates that successful execution largely depends on the judoka’s skill in generating and controlling torque and angular momentum [33]. The effectiveness of this technique is determined by the judoka’s body positioning, grip, and timing, which are crucial for optimizing the force exerted during the throw [34]. Our data indicates that there are no significant differences overall in the velocity and acceleration at which uchi-mata is performed. However, Specialists demonstrate greater displacement by hip during the Approach phase, leading to differences in range of motion (ROM). These findings align with previous studies in combat sports, particularly concerning the hip joint [38,39]. A possible explanation for the limited ROM at higher levels suggests that muscle-tendon units may not efficiently store and release the elastic energy generated during high-intensity movements, such as accelerations, directional changes, and jumping tasks [40]. In this sense, the ROM superiority observed in specialists may reflect not only joint flexibility but also more efficient neuromuscular recruitment patterns, consistent with the concept of motor efficiency reported in other high-performance sports [41]. These differences are also reflected in the positioning of the attacking leg after initiating the movement. These results underscore the importance of this phase in the overall performance of the technique, as athletes begin the movement from a static position. In a study with Taekwondo athletes, Wasik et al. [42] observed that the velocity with which the athlete lifts their foot off the ground is associated (r = 0.61) with the maximum velocity of the an chagi kick.

Previous studies have highlighted that the biomechanics of uchi-mata require precise execution to effectively utilize the opponent’s body weight and maintain balance during the attack [43,44]. Lee et al. [43] identified key factors such as hip rotation, knee flexion, and the timing of lower limb movements which contribute to the throw’s effectiveness. They suggest that optimal execution demands a balance of flexibility and strength, especially in the hip and knee joints, to achieve the necessary ROM and attack power. Our data further supports the critical role of dynamic flexibility combined with strength in the adequate performance of this technique. For example, specialists achieved an average knee height that was 12 cm higher and a foot displacement 16 cm further in the final movement phase (with the confidence interval suggesting this difference could reach 30 cm). These findings align with Suarez et al. [45], who analyzed uchi-mata attempts by 12 high-level Spanish athletes during competitions, and only focusing on successful ippon outcomes. Their study concluded that the most effective throws resulted from significant knee and foot displacement along the Z-axis.

However, it is important to note that the knee and foot height are also influenced by the trunk’s movement toward the supporting limb. Specialists positioned their hips approximately 23 cm lower during the Throw phase (Figure 3). Our findings align with those of Lee et al. [43], who, when comparing 12 experts and 12 beginners, emphasized that the center of mass and the attacking limb positioning are crucial for the efficiency of this technique. Additionally, a study by Lee et al. [35] found that athletes with longer limbs were more efficient in executing uchi-mata, as they demonstrated shorter attack times. Nonetheless, no significant differences in lower limb length were observed between the two groups in our study (Table 1). Taken together, our data suggest that judokas aiming to become proficient in uchi-mata should focus on improving anteroposterior dynamic flexibility.

The global velocity analysis revealed important patterns that were not apparent in phase-specific comparisons. Although hip angular velocity and linear foot velocity approached statistical significance, both showed small to moderate effect sizes, indicating consistent velocity advantages for specialists throughout the entire execution of the technique. These findings are consistent with previous biomechanical analyses of uchimata, which demonstrated greater leg angular velocity, center of mass velocity, and trunk angular momentum in skill compared with less skilled judokas [18,46]. The persistence of velocity advantages despite equivalent acceleration profiles supports the concept of a “velocity reserve” in expert athletes [47]. This phenomenon, also observed in endurance and sprint disciplines [48], suggests that specialists can sustain higher execution velocities without requiring greater instantaneous force production, potentially offering energetic efficiency in competitive settings where repeated throws are required. This trend supports the concept of a “velocity reserve” in expert athletes, whereby higher movement velocities are sustained across all phases rather than being confined to specific components of the technique [47]. Furthermore, studies comparing performance levels in judo athletes indicate that superior efficiency is not necessarily related to greater maximal force, but rather to enhanced neuromuscular coordination, faster rate of force development, and greater movement power [30,31,49]. The absence of acceleration differences, combined with these velocity advantages, therefore suggests that specialists achieve superior movement efficiency primarily through refined neuromuscular control rather than higher force production capacity.

The global movement analysis has important implications for training methodology and talent identification in judo. The finding that displacement parameters consistently showed the largest effect sizes across variables suggests that movement amplitude training, such as enhancing hip and leg range of motion, should be prioritized in uchi-mata development programs. This aligns with biomechanical analyses demonstrating that elite judokas exhibit higher peak angular velocities of the limbs and trunk, superior rotational control, and more effective lower-body movement patterns compared to novices [16]. Additionally, improvements in flexibility have been directly linked to enhanced judo-specific performance: youths undergoing pre-competition training demonstrated increases in flexibility (by approximately 11.5%) coupled with improvements in jumping power, velocity, and fitness as measured by the Special Judo Fitness Test [50]. The consistent velocity advantages observed in specialists, despite similar acceleration capabilities, further indicate that training programs should emphasize movement efficiency and neuromuscular coordination, rather than focusing exclusively on strength or power development. Therefore, our results reinforce that flexibility and ROM are not ancillary qualities but central determinants of uchi-mata success. Integrating dynamic flexibility training with neuromuscular coordination exercises may represent a more effective development pathway than focusing solely on maximal strength or power.

The current experimental protocol has both strengths and limitations. Importantly, sex was included as a fixed effect in the statistical models. However, no significant main effects or interactions with group or phase were observed, indicating that the kinematic differences identified between specialists and non-specialists were consistent across male and female judokas. While this strengthens the generalizability of our findings, we recommend that future studies perform sex-stratified analyses to explore potential biomechanical nuances that may emerge in larger samples. Furthermore, we encounter challenges in comparing results when reviewing the existing literature on this technique as there are few studies, many of which are case studies [21,34,51,52]. Thus, our study introduces a methodological innovation by conducting a comparison of international-level athletes, advancing beyond previous studies that primarily compared experts to beginners [4,5,12]. In addition, our results must be interpreted considering the limitations of our protocol. Since the participants was composed of male and female athletes, since there are biomechanical differences between sex, we recommend that future studies analyze male and female separately. All techniques were performed only by dominant side. Furthermore, we conducted a laboratory analysis, it is important to note that previous studies have shown differences when techniques are applied in competition [53]. It should also be considered that uchi-mata has variations such as the flamingo [54] and koshi-uchi-mata [35], but no comparisons were made between different variations in our study. Moreover, we only analyzed the limb kinematics, while acknowledging that other key actions, such as trunk rotation, are essential for completing the Throw phase [33,34]. Additionally, while the global movement analysis provided valuable insights into overall movement characteristics, it may have obscured phase-specific adaptations that are crucial for technique effectiveness. The aggregation of data across phases assumes equal importance of all technique components, which may not reflect the actual biomechanical demands of competitive uchi-mata execution. Future research should consider weighted global analyses that account for the relative importance of different phases in technique success. However, we believe this limitation does not invalidate the study’s results. The primary factors influencing the technical efficiency of uchi-mata are likely to be found in the lower limbs [34].

### Practical Application

The technical differences identified in this study, particularly the greater hip displacement in the Approach phase and the higher knee and foot positioning in the Throw phase, indicate that targeted training should focus on improving lower limb range of motion, dynamic flexibility, and specific strength. For more information, please see the Supplementary Materials Figure S1.

## 5. Conclusions

Our study directly addressed the research objectives by comparing uchi-mata execution across its three phases between specialist and non-specialist international athletes, identifying kinematic predictors of technique effectiveness and determining whether expertise differences are consistent throughout the movement pattern. Our findings indicate that uchi-mata expertise is characterized primarily by greater movement amplitude and velocity control, rather than maximal power generation. While acceleration profiles were similar between groups, specialists exhibited significantly greater knee displacement and trends toward higher hip angular and linear foot velocities, demonstrating that technical mastery relies on optimized range of motion, refined neuromuscular coordination, and efficient velocity management. These results confirm our hypothesis that specialists differ from non-specialists in both phase-specific and global movement characteristics. In practical terms, these insights highlight the importance of designing training programs that prioritize coordination, range of motion, and velocity control to enhance uchi-mata performance, offering a clear guide for coaches and athletes aiming to optimize competitive outcomes.

## Figures and Tables

**Table 1:** Age, training and anthropometric characteristics of participants.

	Specialist (n = 20)	Non-Specialist (n = 20)	F_calc._; *p*	95%CI for Difference
Sex (♀/♂)	4/16	8/12	-	-
Dominance (R/L)	18/2	17/3	-	-
Age (years)	25.8 ± 6.4	22.9 ± 5.3	2.428; 0.127	2.9 (−0.9;6.7)
Body mass (kg)	68.5 ± 9.6	67.9 ± 15.0	0.64; 0.802	0.6 (−7.4; 8.7)
Height (cm)	168.1 ± 7.9	168.8 ± 9.5	0.024; 0.877	0.0 (−0.1; 0.1)
BMI (kg/m^2^)	24.2 ± 1.9	23.6 ± 3.4	0.419; 0.521	0.6 (−1.2; 2.3)
LL height (cm)	87.4 ± 4.0	88.5 ± 5.2	0.369; 0.547	0.9 (−3.9; 2.1)
Training (h/wk)	18.8 ± 1.1	18.8 ± 2.3	0.01; 1.0	0.6 (−1.1; 1.1)

kg—kilograms. cm—centimeters. BMI—Body mass index. kg/m^2^—kilograms per meter squared. LL—lower limbs. h/wk—hours per week. *p*—statistics calculated. CI—confidence interval. R—right. L—left.

**Table 2:** Kinematic analysis for uchi-mata performed by the groups.

	Specialists	Non-Specialists	F Calc; *p* Value	95%CI for Difference	Cohen’s d
	Time (s)				
Approach	0.5 ± 0.2 ^a^	0.4 ± 0.1	4.73; 0.035	0.1 (0.01; 0.2)	0.687
Turning	0.2 ± 0.1	0.3 ± 0.1	0.593; 0.446	−0.07 (−0.25; 0.11)	0.243
Throw	0.6 ± 0.1 ^a^	0.5 ± 0.1	7.25; 0.01	0.09 (0.02; 0.16)	0.851
	Angular hip velocity (°/s)				
Approach	137.3 ± 50.2	118.5 ± 38.2	1.773; 0.191	18.8 (−9.76; 47.38)	0.421
Turning	119.4 ± 56.0	110.4 ± 52.5	0.276; 0.603	9.01 (−25.74; 43.76)	0.166
Throw	179.3 ± 65.1	149.2 ± 55.1	2.501; 0.122	30.15 (−8.45; 68.75)	0.5
	Angular knee velocity (°/s)				
Approach	207.6 ± 86.8	217.0 ± 91.3	0.112; 0.74	−9.42 (−66.43; 47.59)	0.1
Turning	330.3 ± 126.8	296.1 ± 87.9	0.979; 0.329	34.15 (−35.71; 104.02)	0.312
Throw	167.6 ± 87.2	161.0 ± 127.7	0.037; 0.849	6.62 (−63.39; 76.63)	0.06
	Linear foot velocity (m/s)				
Approach	1.5 ± 0.9	1.3 ± 0.9	0.647; 0.426	0.23 (−0.35; 0.82)	0.254
Turning	3.0 ± 1.2	2.6 ± 1.0	1.819; 0.185	0.46 (−0.23; 1.15)	0.426
Throw	2.2 ± 0.8	1.8 ± 0.4	3.36; 0.075	0.38 (−0.04; 0.8)	0.579
	Angular hip acceleration (°/s^2^)				
Approach	1899.1 ± 1008.5	2136.3 ± 1052.2	0.215; 0.645	−237.24 (−1,272.32; 797.83)	0.146
Turning	2584.1 ± 1503.2	2420.2 ± 1292.5	0.137; 0.714	163.89 (−733.52; 1061.29)	0.116
Throw	2841.7 ± 1285.0	3152.8 ± 1463.6	0.251; 0.619	−311.03 (−1568.82; 946.76)	0.158
	Angular knee acceleration (°/s^2^)				
Approach	3803.5 ± 2136.0	4267.9 ± 2118.4	0.4776 0.494	−464.39 (1826.15; 897.39)	0.218
Turning	6223.9 ± 4113.9	5047.3 ± 2127.0	1.291; 0.263	1176.61 (−919.82; 3273.03)	0.359
Throw	3758.9 ± 2656.5	4860.4 ± 2219.4	0.707; 0.406	−1101.56 (−3752.63; 1549.51)	0.266
	Linear foot acceleration (m/s^2^)				
Approach	18.7 ± 10.5	19.6 ± 10.6	0.049; 0.825	−0.94 (−9.48; 7.6)	0.09
Turning	26.5 ± 9.3	25.6 ± 10.3	0.072; 0.789	0.83 (−5.45; 7.12)	0.09
Throw	17.1 ± 5.2	15.6 ± 3.9	0.997; 0.324	1.45 (−1.49; 4.38)	0.33
	Hip displacement (m)				
Approach	0.3 ± 0.1 ^a^	0.2 ± 0.1	7.926; 0.007	−0.08 (−0.15; −0.02)	1.0
Turning	0.3 ± 0.1	0.2 ± 0.1	0.771; 0.385	0.02 (−0.03; 0.08)	1.0
Throw	0.1 ± 0.1	0.1 ± 0.1	0.505; 0.481	0.01 (−0.02; 0.04)	0.001
	Knee displacement (m)				
Approach	0.4 ± 0.1	0.4 ± 0.2	0.538; 0.467	0.04 (−0.07; 0.15)	0.07
Turning	0.5 ± 0.1	0.5 ± 0.1	0.049; 0.825	0.01 (−0.08; 0.09)	0.085
Throw	0.5 ± 0.2 ^a^	0.4 ± 0.1	9.0; 0.047	−0.16 (−0.27; −0.05)	0.315
	Foot displacement (m)				
Approach	0.5 ± 0.2	0.5 ± 0.3	0.747; 0.392	0.07 (−0.09; 0.22)	0.89
Turning	0.7 ± 0.2	0.7 ± 0.3	0.091; 0.763	−0.02 (−0.18; 0.13)	0.277
Throw	1.1 ± 0.2 ^a^	0.9 ± 0.2	10.082; 0.002	−0.23 (−0.38; −0.08)	0.224

Note: s—seconds. °/s—degrees per second. m/s—meters per second. °/s^2^—degrees per second squared. m/s^2^—meters per second squared. m—meters. ^a^ *p* ≤ 0.05 vs. non-specialist. *p*—statistics calculated. CI—confidence interval.

## Data Availability

Data from this study can be requested directly from the corresponding author.

## Supplementary Materials

The following supporting information can be downloaded at: https://www.mdpi.com/article/10.3390/jfmk10040378/s1, Figure S1: Complementary specific uchi-mata training.

## Abbreviations

The following abbreviations are used in this manuscript:

BMI	Body mass index
LLi	Lower limbs
CI	Confidence interval
Dh	Hip displacement
Dk	Knee displacement
DF	Foot displacement
NSC	Non-standardized coefficient
SC	Standardized coefficient
LL	Lower limit
UL	Upper limit
Sig	Significance
