# Supplementary material for: Kinematic Analysis of the Lower Limb in Uchi-Mata: Comparison Between Elite Athletes Specializing and Non-Specializing
Source: J Funct Morphol Kinesiol. 2025 Sep 30;10(4):378. doi: 10.3390/jfmk10040378 (PMC12551108; doi:10.3390/jfmk10040378)
Supplement: Supplementary file 1 [file jfmk-10-00378-s001.zip › jfmk-3767541-supplementary.pdf]

Supplementary material. Suggestion of specific *uchi-mata* training

Based on our findings, a complementary training sequence is proposed to optimize *uchi-mata* performance (Suppl. Figure S1). This sequence emphasizes progressive development of flexibility, limb mobility, coordination, and strength, integrating these elements across the three phases of *uchi-mata* (Approach, Turning, and Throw) to enhance technical execution. It is important to note that these exercises are conceptual recommendations derived from the study results and are intended to guide training design rather than represent validated or previously tested protocols.

The sequence begins with anteroposterior static stretching (4 sets of 30 seconds, with 30 seconds rest) to increase flexibility and facilitate optimal attack positioning. Next, anteroposterior attack limb movements are performed, both forward and backward, to develop mobility and coordination (4 sets of 10 repetitions, 1 minute rest).

To enhance strength and control, back limb attacks using elastic resistance are included, starting from the initial position to the final attack position (4 sets of 10 repetitions, 1 minute rest). This is followed by the Throw phase using judogi handgrip with spring or elastic resistance, executed from start to finish (4 sets of 10 repetitions, 1 minute rest).

The progression continues with the complete *uchi-mata* using elastic resistance, performed in three phases, Approach, Turning, and Throw (4 sets of 10 repetitions, 1 minute rest). Finally, the complete *uchi-mata* with judogi handgrip and spring resistance is executed, also in three phases, to replicate match-like conditions and resistance patterns (4 sets of 10 repetitions, 1 minute rest). This structured progression aims to combine mobility, strength, and technical refinement in a single integrated program, directly targeting the kinematic variables found to differentiate specialists from non-specialists.

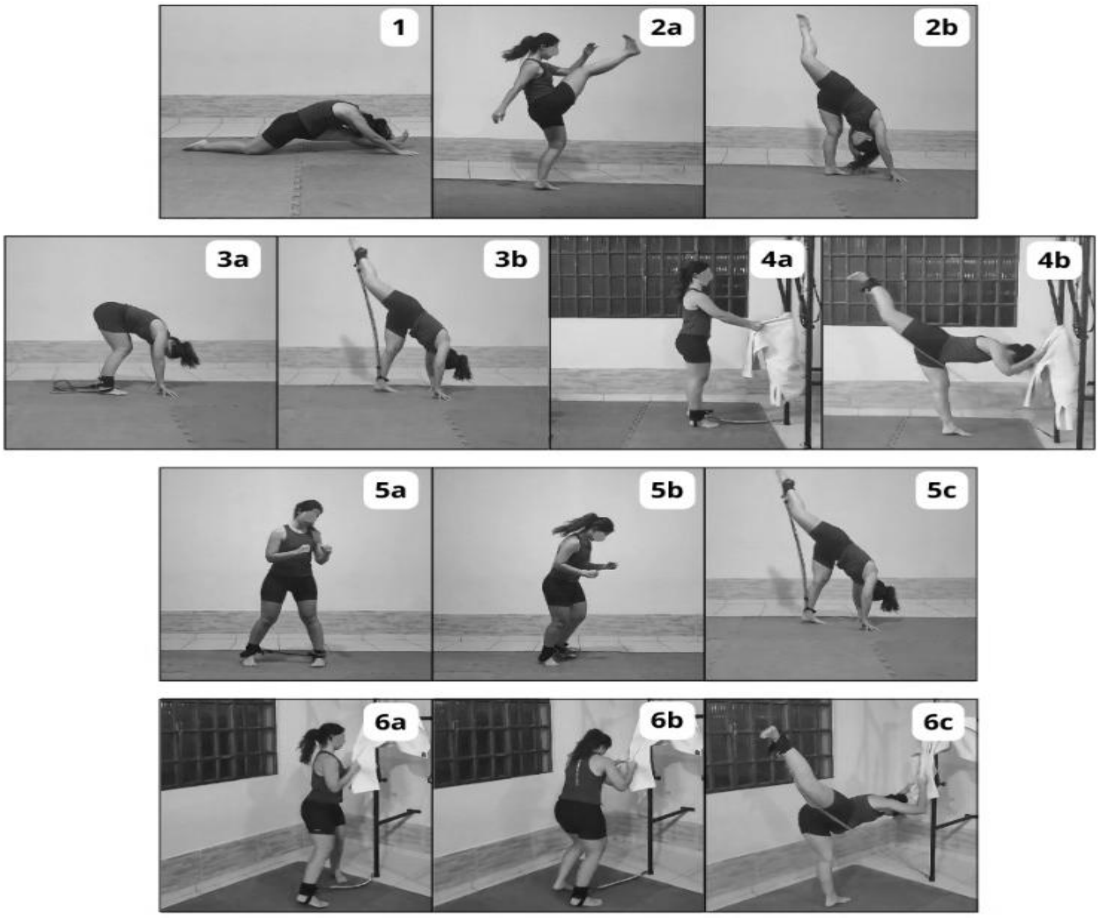

Suppl. Figure S1. Complementary specific *uchi-mata* training:
